# Machine learning assisted Cameriere method for dental age estimation

**DOI:** 10.1186/s12903-021-01996-0

**Published:** 2021-12-15

**Authors:** Shihui Shen, Zihao Liu, Jian Wang, Linfeng Fan, Fang Ji, Jiang Tao

**Affiliations:** 1 Department of General Dentistry, Shanghai Ninth People’s Hospital, Shanghai Jiao Tong University School of Medicine; College of Stomatology, Shanghai Jiao Tong University; National Center for Stomatology; National Clinical Research Center for Oral Diseases, Shanghai Key Laboratory of Stomatology, Shanghai, People’s Republic of China; 2 Department of Nuclear Medicine, Xin Hua Hospital Affiliated to Shanghai Jiao Tong University School of Medicine, Shanghai, People’s Republic of China; 3 Department of Orthodontics, Shanghai Ninth People’s Hospital, Shanghai Jiao Tong University School of Medicine; College of Stomatology, Shanghai Jiao Tong University; National Center for Stomatology; National Clinical Research Center for Oral Diseases, Shanghai Key Laboratory of Stomatology, Shanghai, People’s Republic of China; 4 Department of Radiology, Shanghai Ninth People’s Hospital, Shanghai Jiao Tong University School of Medicine; College of Stomatology, Shanghai Jiao Tong University; National Center for Stomatology; National Clinical Research Center for Oral Diseases, Shanghai Key Laboratory of Stomatology, Shanghai, People’s Republic of China

**Keywords:** Machine learning, Dental age, Tooth development, Cameriere

## Abstract

**Background:**

Recently, the dental age estimation method developed by Cameriere has been widely recognized and accepted. Although machine learning (ML) methods can improve the accuracy of dental age estimation, no machine learning research exists on the use of the Cameriere dental age estimation method, making this research innovative and meaningful.

**Aim:**

The purpose of this research is to use 7 lower left permanent teeth and three models [random forest (RF), support vector machine (SVM), and linear regression (LR)] based on the Cameriere method to predict children's dental age, and compare with the Cameriere age estimation.

**Subjects and methods:**

This was a retrospective study that collected and analyzed orthopantomograms of 748 children (356 females and 392 males) aged 5–13 years. Data were randomly divided into training and test datasets in an 80–20% proportion for the ML algorithms. The procedure, starting with randomly creating new training and test datasets, was repeated 20 times. 7 permanent developing teeth on the left mandible (except wisdom teeth) were recorded using the Cameriere method. Then, the traditional Cameriere formula and three models (RF, SVM, and LR) were used to estimate the dental age. The age prediction accuracy was measured by five indicators: the coefficient of determination (R^2^), mean error (ME), root mean square error (RMSE), mean square error (MSE), and mean absolute error (MAE).

**Results:**

The research showed that the ML models have better accuracy than the traditional Cameriere formula. The ME, MAE, MSE, and RMSE values of the SVM model (0.004, 0.489, 0.392, and 0.625, respectively) and the RF model (− 0.004, 0.495, 0.389, and 0.623, respectively) were lower with the highest accuracy. In contrast, the ME, MAE, MSE and RMSE of the European Cameriere formula were 0.592, 0.846, 0.755, and 0.869, respectively, and those of the Chinese Cameriere formula were 0.748, 0.812, 0.890 and 0.943, respectively.

**Conclusions:**

Compared to the Cameriere formula, ML methods based on the Cameriere’s maturation stages were more accurate in estimating dental age. These results support the use of ML algorithms instead of the traditional Cameriere formula.

**Supplementary Information:**

The online version contains supplementary material available at 10.1186/s12903-021-01996-0.

## Introduction

The age estimation of teeth plays an important role in both clinical and forensic medicine. Many studies have investigated various methods to accurately estimate age in both living and deceased individuals, especially in children and adolescents. An accurate estimation of age is crucial, as it can be applied to determine the appropriate sentencing time and treatment strategy [1, 2]. Sophisticated medical techniques widely applied to age estimation include analyzing skeletal maturity or dental development [3, 4]. However, the chronic diseases or nutritional deficiencies that an individual experienced during their growth and development may result in age estimate deviations. Compared to skeletal maturity, tooth development is less affected by environmental factors [5, 6], which may be related to the strict genetic control of tooth development [7].

The Demirjian method uses 8 stages, A to H, to classify teeth based on maturity and calcification [8]. Willems et al. modified the Demirjian method and then provided a new method of scoring that allows a direct conversion from classification to age [9]. A study on the application of the Demirjian and Willems methods in 7- to 14-year-old adolescents in China revealed that neither method was suitable for adolescents in the region [10]. Therefore, a more accurate method needs to be investigated.

Recently, a new method developed by Cameriere has been widely recognized and well accepted [11–20]. Cameriere established a European formula by gauging open apices of 7 permanent teeth on the left mandible of a panoramic radiograph [11]. The Demirjian method has a certain degree of subjectivity that leads to a relatively high level of personal error. Similarly, the application of the Cameriere method warrants adequate experience to minimize errors. In 2015, Guo et al. used the Cameriere method to propose a formula for estimating dental age in China [21].

Currently, machine learning has been providing a welcome boost to the estimation of bone age [22, 23] and the process of its utilization for dental age is gradually accelerating [24–26]. As a fundamental approach of artificial intelligence, machine learning enables us to predict dental age not only more accurately but also more efficiently. Machine learning methods are more accurate for describing than traditional radiology methods [24–26]. In the work by Tao et al., a supervised machine learning method is employed using a statistical model created by optimizing the model derived from the “known” data set [25]. Tao and Galibourg applied machine learning to the Demirjian and Willams method for dental age estimation [24, 25]. However, these recent studies are not based on the Cameriere method for dental age estimation.

The purpose of this research is to use three models (random forest, support vector machine, and linear regression) to predict children's dental age based on the Cameriere method, using 7 lower left permanent teeth, and comparing the methods with the Cameriere formula.

## Materials and methods

### Samples

All methods were carried out in accordance with relevant guidelines and regulations. The research was authorized by the Independent Ethics Committee of the Shanghai Ninth Hospital affiliated with Shanghai Jiao Tong University, School of Medicine (2017-282-T212). The research content does not involve patient privacy, so informed consent was obtained from all subjects or their legal guardian(s) before undergoing imaging examinations and participating in the study.

This retrospective study selected digital panoramic radiographs taken by a KODAK 8000C Panoramic and Cephalometric Digital Dental X-ray Machine collected during outpatient treatment between 2000 and 2013. A total of 748 children and adolescents in Eastern China were included in the study (Table 1)**,** distributed according to age and sex. The chronological ages of these children and adolescents were definite. In total, 356 females and 392 males aged 5.00 to 13.99 years were evaluated. Digital orthopantomograms (OPGs) were divided into 9 sets based on the chronological age of each subject. The first group comprised subjects aged between 5.00 and 5.99 years, the second group comprised subjects aged 6, and so on. According to the guidelines provided by Schmeling et al. [27], we tried to make the subjects in age groups evenly distributed, the number of boys and girls was balanced, and for most groups, at least the number of subjects was 10 times the number of direct examination features (7 permanent teeth and sex, which are the 8 direct characteristics examined in this study).

**Table 1:** Age groups and gender distribution in Eastern China sample, respectively

Age group	Gender		Total
	Female	Male	
5.00–5.99	20	18	38
6.00–6.99	47	45	92
7.00–7.99	35	44	79
8.00–8.99	52	42	94
9.00–9.99	48	63	111
10.00–10.99	44	48	92
11.00–11.99	45	43	88
12.00–12.99	35	45	80
13.00–13.99	30	44	74
Total	355	392	748

The inclusion criteria of OPGs were as follows: no lack of mandibular first premolar; no more hyperdontia; no systemic disease; no history of root canal treatment for the mandibular first premolar; clear or high-quality panorama OPGs; and no related diseases affecting jaw development, such as cysts or cancer.

### Radiographic evaluation

**Fig. 1:**
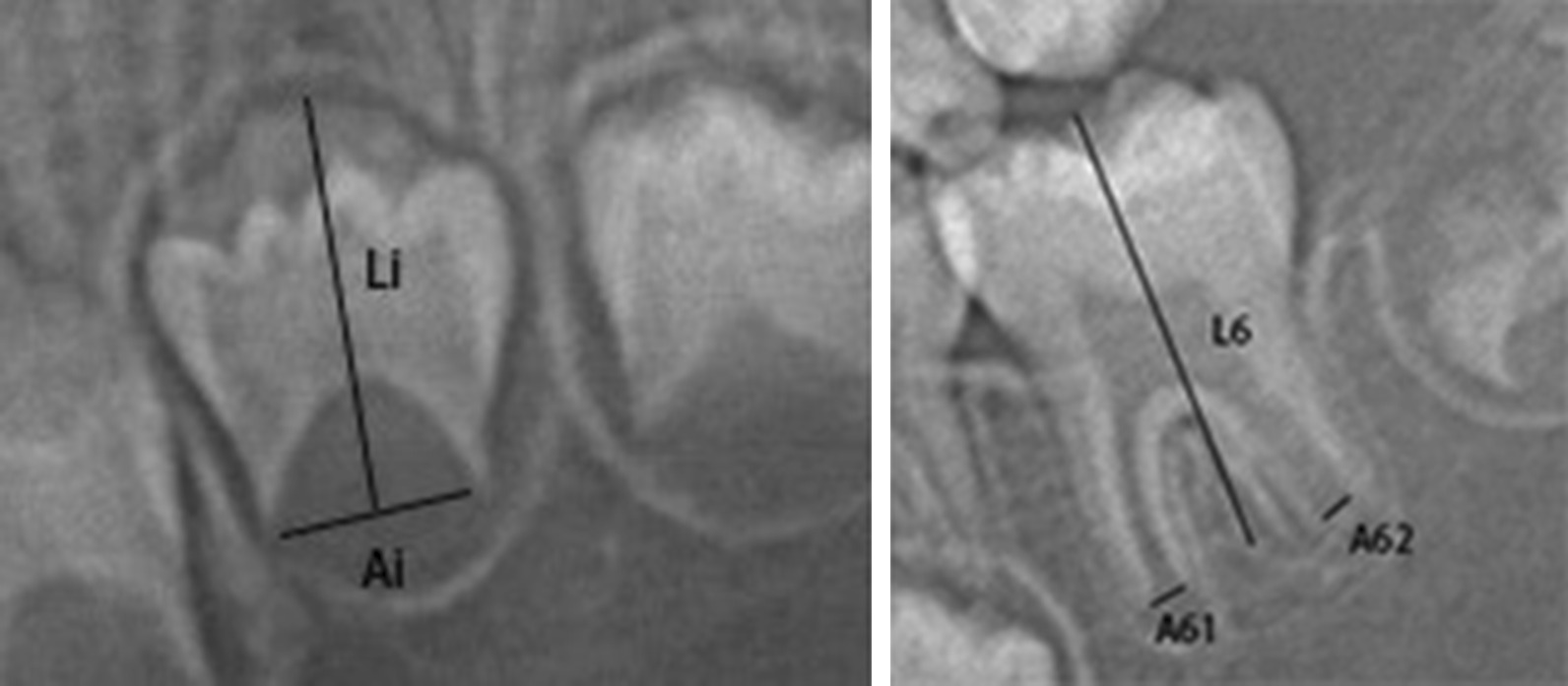
Left: An example of single root tooth measurement. A_i_, i = 1, …,5 (teeth with one root), is the distance between the inner sides of the open apex; Right: An example of multiple root tooth measurement. A_i_, i = 6,7 (teeth with two open apices) is the sum of the distances between the inner sides of the two open apices; and L_i_, i = 1,…,7, is the length of the seventh teeth

Digital OPGs were stored on a computer and processed using computer-aided measuring software (Adobe Photoshop CC 2017). Object detection algorithms were not needed in this study. All the relevant digital OPG data were measured by two observers, and machine learning was performed on this basis. The Cameriere method has previously been applied for dental age estimation [11–20]. In brief, 7 permanent developing teeth on the left mandible (except wisdom teeth) were recorded, including the number of apical ends with completely closed roots (N_0_). The distance between the inner side of the open apices (A_i_, i = 1, …,7) was measured. Considering the amplification and angulation effects caused by possible differences in the OPGs, measurements were divided by the tooth length (L_i_, i = 1, …,7) to obtain normalized measurements (Fig. 1). If the development of the tooth was complete and the apical foramen was completely closed, then X_i_ (i = 1, …,7) = 0; otherwise, X_i_ (i = 1, …,7) was calculated by dividing the distance between the apical fora and by tooth length (X_i_ = A_i_/L_i,_ i = 1, …,7).

### Age estimation method

The machine learning models were trained on the information sources as follows: sex (g), the normalized measurements of the 7 permanent developing teeth on the left mandible (X_i_, i = 1, …,7), the sum of the normalized open apices (s, s = X_1_ + X_2_ + ⋯ + X_7_), the number of teeth with complete root development (N_0_) and the first-order relationship between s and N_0_ (s·N_0_). The target value was the chronological ages.

The following machine learning supervised regression algorithms were tested: random forest (RF), support vector machine (SVM) and linear regression (LR) model. The tuning of the hyperparameters to obtain the best model was achieved by exploring multiple combinations using the GridSearchCV function and K-fold cross-validation [28, 29]. Briefly, image data were divided into K groups, and (K − 1) groups were used as training data, and one data group was used for validation. This process was repeated K times until each of the K groups became a validation dataset. The number of groups (K) was calculated using Sturges' formula (K = 1 + log_2_N). Sturges' formula is used to decide the number of classes in the histogram [30, 31]. Thus, in this study, we categorized the data into ten groups. To avoid overfitting, a 20% validation dataset was used during hyperparameter optimization.

To compare the different machine learning models with the two Cameriere formulas, the datasets were randomly divided into a training dataset and test dataset in an 80–20% proportion. The training set was approximately 598 radiographs (80% of 748), and the test set was approximately 150 (20% of 748). The entire procedure, starting with the random creation of new training and test sets, was repeated 20 times.

The hyperparameters described in Additional file 1: Table S1 were tuned.

In addition, we also used Eqs. (1) and (2) derived by Cameriere et al. (European formula) and Guo et al. (Chinese formula), respectively, to estimate the dental age and compared the results with the three machine learning models [11, 32].

1 $${\text{Age}} = 8.387 + 0.282{\text{g}} - 1.692X_{5} + 0.835N_{0} - 0.116s - 0.139\;s \cdot N_{0}$$

2 $${\text{Age}} = 10.202 + 0.826{\text{g}} - 4.068X_{3} - 1.536X_{4} - 1.959X_{7} + 0.536N_{0} - 0.219s \cdot N_{0}$$

### Statistical analyses

The accuracy of age prediction was measured by five indicators: the coefficient of determination (R^2^), mean error (ME; chronological age minus dental age), root mean square error (RMSE), mean square error (MSE), and mean absolute error (MAE). These four indicators can be obtained using Eq. (3).

3 $$\begin{aligned} & ME = \frac{1}{n}\mathop \sum \limits_{i = 1}^{n} p_{i} - e_{i} \\ & MAE = \frac{1}{n}\mathop \sum \limits_{i = 1}^{n} \left| {p_{i} - e_{i} } \right| \\ & MSE = \frac{1}{n}\mathop \sum \limits_{i = 1}^{n} \left( {p_{i} - e_{i} } \right)^{2} \\ & RMSE = \sqrt {\frac{1}{n}\mathop \sum \limits_{i = 1}^{n} \left( {p_{i} - e_{i} } \right)^{2} } \\ \end{aligned}$$

In Eq. (3), p_i_ represents the predicted value, e_i_ represents the expected value, and n is the total number of sample points. The RMSE, MSE, MAE, and R^2^ are often used to assess the accuracy of model predictions [33]. The smaller the RMSE, MSE, and MAE are, the higher the accuracy of the predictions is. The larger the value of R^2^ is, the better the fit. The ME was calculated to quantify the direction of the error, where a positive value indicated that the dental age was underestimated, and a negative number indicated the opposite. The MAE was calculated to quantify the magnitude of the error.

**Fig. 2:**
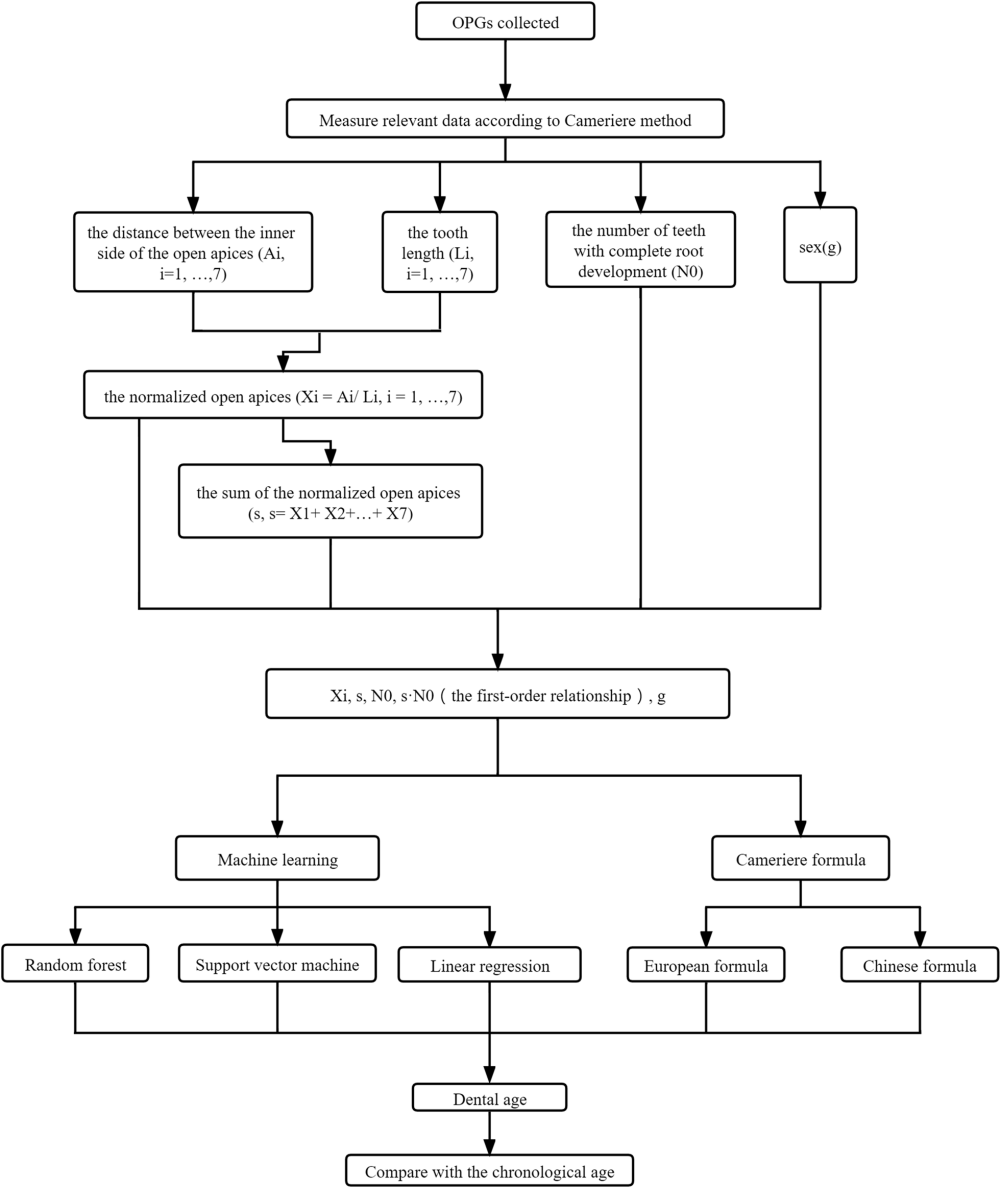
A schematic outline of the operational procedures and analytical steps

Figure 2 shows an outline of the operational procedures and analytical steps.

All experiments were performed using SPSS 25.0 (IBM Corp. Released 2017. IBM SPSS Statistics for Windows, Version 25.0. Armonk, NY: IBM Corp.), the ScikitLearn 0.24.2 libraries [34] and Python 3.8.2. The significance level was set to 0.05. Scikit-learn 0.24.2 libraries is a free software machine learning library for the Python programming language, which does not have a GUI. Python 3.8.2 is a programming language. SPSS 25.0 was used for the statistics of Cameriere's formula.

### Intra and interobserver agreement

The 2 observers who participated in the study were trained in the age estimation method. Each observer assessed each of the 748 radiographs. To assess the reliability between the observers, all observers twice evaluated 50 randomly selected X-rays before starting the original study. The intraclass correlation coefficient (ICC) for the intraobserver agreement was 0.93 for both observers, whereas it was 0.91 for the interobserver agreement. The results of the intraclass correlation coefficient showed interobserver and test–retest reliability.

## Results

**Fig. 3:**
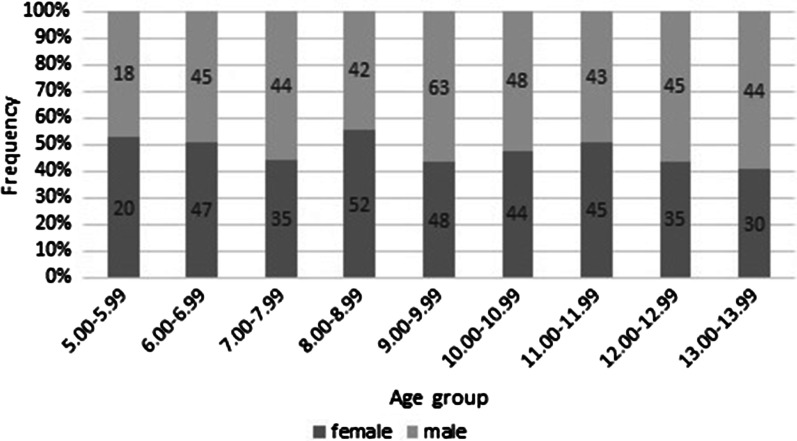
Age and sex distribution for each category of age per year

A total of 748 panoramas (356 females and 392 males) were included in this study. The age and sex distribution of the datasets are plotted in Fig. 3. The distribution of males and females in each group was relatively even.

**Fig. 4:**
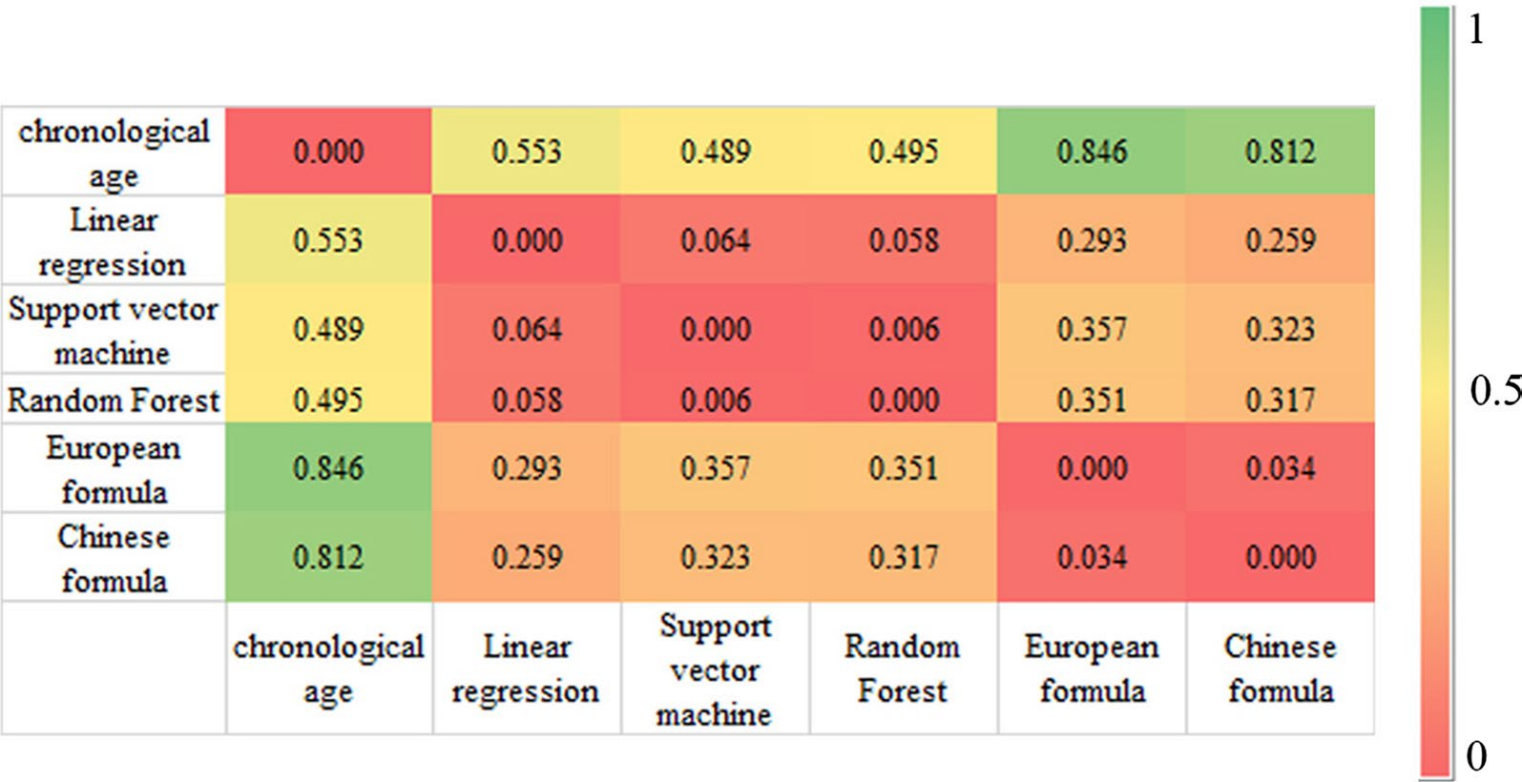
Heat map showing the mean of the mean absolute errors (MAE) calculated from the 20 replicates for each pair of dental age estimation methods

The dental age prediction results are shown in Table 2. All tested machine learning methods were significantly more accurate than the two Cameriere formulas for all metrics (Fig. 4).

**Table 2:** Mean error (ME), mean absolute error (MAE), mean square error (MSE), root mean square error (RMSE), and R^2^ values assessing performance of machine learning regression methods and Cameriere European/Chinese formula for chronological age estimation

Method	ME ± SD	MAE ± SD	MSE ± SD	RMSE ± SD	R^2^ ± SD
Linear regression	0.008 ± 0.052 (− 0.095–0.094)	0.553 ± 0.026 (0.501–0.589)	0.488 ± 0.063 (0.396–0.588)	0.698 ± 0.045 (0.629–0.767)	0.909 ± 0.012 (0.890–0.925)
Support vector machine	0.004 ± 0.063 (− 0.142–0.104)	0.489 ± 0.030 (0.422–0.552)	0.392 ± 0.049 (0.286–0.480)	0.625 ± 0.039 (0.535–0.693)	0.925 ± 0.011 (0.900–0.949)
Random Forest	− 0.004 ± 0.046 (− 0.090–0.088)	0.495 ± 0.024 (0.446–0.533)	0.389 ± 0.039 (0.309–0.461)	0.623 ± 0.032 (0.556–0.679)	0.928 ± 0.009 (0.914–0.945)
European formula	0.592 ± 0.032 (0.532–0.654)	0.846 ± 0.228 (0.801–0.891)	0.755 ± 0.038 (0.684–0.829)	0.869 ± 0.022 (0.827–0.911)	–
Chinese formula	0.386 ± 0.035 (0.322–0.450)	0.812 ± 0.022 (0.530–0.655)	0.890 ± 0.049 (0.796–0.997)	0.943 ± 0.026 (0.892–0.999)	–

**Fig. 5:**
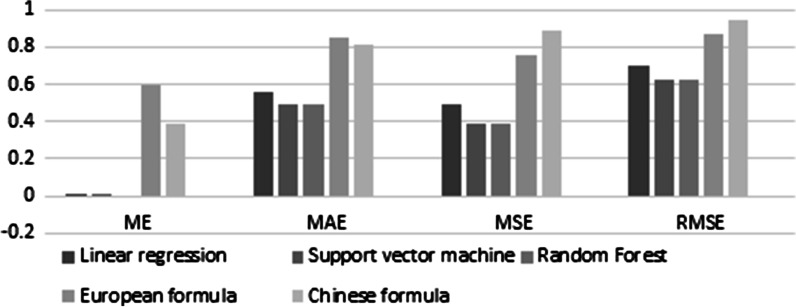
ME, MAE, MSE and RMSE of machine learning methods (LR, SVM & RF) and Cameriere formula (European, Chinese formula)

It can be seen from Fig. 5 that compared with the Cameriere European or Chinese formula, all the LR model, SVM model, and RF model can greatly reduce the ME. In addition, the MAE, MSE, and RMSE all have different degrees of reduction, which means that the machine learning algorithms we evaluated have indeed improved the accuracy of predicting the children’s dental age.

Regarding the ME, the SVM model (0.004) and the RF model (− 0.004) performed best. The former estimated average dental age underestimated 0.004 years, and the latter estimated average dental age overestimated 0.004 years. For the MAE, the SVM model has the lowest MAE (0.489); that is, the dental age estimated by the SVM model is the closest to the chronological age, and the MAE value is 0.489 years. The RF model has the lowest MSE, which is 0.389 years old. In general, among the three models, the SVM model and the RF model have the highest accuracy.

## Discussion

Since the estimation of the age of children and adolescents through teeth is important in forensic medicine and clinical practice, we chose subjects aged 5–13 years. It is vital for athletes to be tested using age estimation to determine their eligibility to compete [35]. For children whose birth dates are not clear, such as those lost or trafficked, it is also important to determine their age. This is the first study of dental age estimation on the optimization of the Cameriere method using machine learning algorithms.

We first use the Cameriere European and Chinese formulas to test our samples. The dental age obtained by the European formula is underestimated by 0.690 years for males and 0.484 years for females; the dental age obtained using the Chinese formula is underestimated by 0.486 years for males and 0.275 years for females. The results of the European formula are basically the same as those in other parts of the world (German males are underestimated by 0.07 years, females by 0.08 years [18]; Turkey underestimates by 0.24 years for females and 0.47 years for males [15]). The local population has an influence on the estimation of dental age. The Cameriere formula of a region is more suitable for people who are geographically close.

The research results using the Cameriere European and Chinese formulas in East China are also consistent with the results of Guo et al.'s research in North China [21]. Guo et al. used the two formulas to infer the age of a sample population in North China. For North China, the dental age inferred using the European formula was underestimated by 0.23 years, and the dental age inferred using the Chinese formula was underestimated by 0.04 years. The accuracy of the Chinese formula is higher than that of European public announcements. It is hoped that more regional samples will be included in future studies.

The second step of our research was to perform machine learning on samples to improve the accuracy of dental age estimation. Regression-based methods aim to find the effect of a set of independent variables on the dependent variable of interest [36]. This is easy but is prone to random errors. In this study, the LR model was carried out in 20 random repeated experiments, but its indicators were still the highest among the three models, that is, the accuracy was lower. In addition, regression methods require a predetermined model for data fitting. The SVM model does not require a predefined model and works in scenarios where there is a high number of variables in comparison to the number of data points [37, 38]. Regarding the R^2^, the SVM model (0.925) was higher than the LR model (0.909). This study proved that the accuracy of the SVM model is high. The RF model decreases the similarity between the individual trees and thus improves the robustness of the final model by selecting the split point at each step in the tree building process from a random subset of the input attributes [39]. In this case, the R^2^ of the RF model is the highest among the three models (0.928).

The study by Tao et al. showed that the MAE and RMSE of the Demirjian method and Willem method inferred dental ages in the Eastern Chinese population aged 11–19 years old are both greater than 1 year (Table 3), which is higher than the European or Chinese Cameriere formulas of this study (Table 2) [25]. Even with the multilayer perceptron, the Demirjian method and Willem method are not as accurate as the European or Chinese Cameriere formulas. This may be caused by the different age ranges of the samples, or it may mean that the Cameriere method is a better choice for inferring the dental age for the Eastern Chinese population. When using machine learning, the SVM model and the RF model based on the Cameriere method reduce the MAE to less than 0.500 and the MSE and RMSE to less than 1.000. This may mean that for children in Eastern China, using the SVM model or the RF model optimized for the Cameriere dental age estimation method can obtain more accurate results.

**Table 3:** RMSE, MSE, MAE values of experimental results using Demirjian’s method (D-method), Willem’s method (W-method), and Multi-layer Perceptron (MLP) by Tao et. al

Male	RMSE	MSE	MAE	Female	RMSE	MSE	MAE
D-method	1.596	2.548	1.307	D-method	1.677	2.812	1.364
W-method	1.602	2.556	1.291	W-method	1.788	3.196	1.407
MLP	1.332	1.775	0.990	MLP	1.617	2.616	1.261

This study confirmed that the SVM model, LR model, and RF model do have better accuracy than the traditional method (Cameriere formula), which is consistent with the results of Galibourg et al., that is, machine learning can significantly improve the accuracy of dental age estimation [24].

Galibourg et al. used machine learning-assisted Demirjian and Willems methods to estimate the dental age of children aged 4–16 [24]. In their research results, the dental age obtained by the SVM model was underestimated by 0.016 years, and the dental age obtained by the RF model was overestimated by 0.007 years, which is similar to the results of this paper (the dental age using the SVM model was underestimated by 0.004 years and using the RF model was overestimated by 0.004 years). Perhaps at this point, we can say that for children under the age of 16, the use of machine learning-assisted dental age estimation can greatly improve the accuracy. This is of great significance to forensic medicine.

In addition to the classic machine learning regression methods used for dental age estimation, Vila-Blanco et al. used deep learning techniques for dental age estimation. The advantage of deep neural networks is that they do not rely on manual measurements or classifications, which require extensive time and effort. The research results of Vila-Blanco et al. showed that for children under 15 years old, the dental age estimated using deep learning is overestimated by 0.07 years [26]. Although deep learning can save time through object detection, its mean error is slightly higher than machine learning regression methods. Compared with manual methods, both deep learning and machine learning regression methods make an average error closer to zero, showing high accuracy.

In this machine learning research, the test set and training set cannot be treated separately. According to studies by Tao and Galibourg et al., a pure test set is not necessary [24, 25]. As datasets, they conformed to the guidelines provided by Schmeling et al. [27]. Through cross-validation, the test set was large to eliminate bias and draw conclusions. This is a study about the difference between estimated age and chronological age, not a classification diagnosis. For this reason, true and false positive/negative values, positive predictive value, negative predictive value, sensitivity, specificity, ROC curves and the success rates of age estimation were not applicable. P values and 95% confidence are based on hypothesis verification. The machine learning methods in this study did not use hypothesis verification, so P values and 95% confidence values cannot be given.

There are some areas for improvement in this study. The sample source is relatively simple, mainly from East China. It is hoped that more regions in the world will conduct machine learning-assisted dental age estimation research.

## Conclusion

In this paper, we found machine learning algorithms improve the accuracy of dental age estimation. Research has shown that the SVM model, the LR model, and the RF model do have better accuracy than the European or Chinese Cameriere formulas. Among all methods, the ME, MAE, MSE, and RMSE values of the SVM model and the RF model are lower and have the highest accuracy. These results support the use of machine learning algorithms instead of using the traditional Cameriere formula.

## Supplementary Information

**Additional file 1: Table S1.** List of the tuned hyperparameters for each Machine Learning algorithm.

## Data Availability

Data sharing is not applicable to this article as no datasets were generated or analysed during the current study.

## Abbreviations

LR: Linear regression
MAE: Mean absolute error
ME: Mean error; chronological age minus dental age
MSE: Mean square error
R^2^: Coefficient of determination
RF: Random forest
RMSE: Root mean square error
SVM: Support vector machine
