# Supplementary material for: Machine learning assisted Cameriere method for dental age estimation
Source: BMC Oral Health. 2021 Dec 15;21:641. doi: 10.1186/s12903-021-01996-0 (PMC8672533; doi:10.1186/s12903-021-01996-0)
Supplement: Supplementary file 1 — Additional file 1: Table S1. List of the tuned hyperparameters for each Machine Learning algorithm. [file 12903_2021_1996_MOESM1_ESM.docx]

Additional file 1: Table S1: List of the tuned hyperparameters for each Machine Learning algorithm. For each hyperparameter, the values inside square brackets were explored by Grid Search.

| Random Forest (RF) | n_estimators: the number of trees in the forest [5, 10, 15, 20, 30, 40, 50],  max_depth: the maximum depth of a tree [6 to 8]  max_features: the number of features to consider when looking for the best split at a node [auto, sqrt, log2]  min_samples_split: the minimum number of samples required to split an internal node [2 to 5]  min_samples_leaf: the minimum number of samples required to be at a leaf node [2 to 5]  bootstrap: whether bootstrap samples are used when building trees [True, False] |
| --- | --- |
| Support Vector Machine (SVM) | kernel: the kernel type used in the algorithm [polynomial, linear, rbf]  gamma: kernel coefficient [0.0001, 0.0005, 0.001, 0.01, 0.05, 0.1]  C: the regularization parameter. The higher the parameter the lower the regularization strength [1 to 10] |
